# A Systematic Review of the Mechanisms and Therapeutic Applications of GHK-Cu (Glycyl-L-Histidyl-L-Lysine-Copper Complex) in Topical Microneedle Delivery: A Case for Expanded Clinical Trials

**DOI:** 10.26502/aimr.0255

**Published:** 2026-08-29

**Authors:** Niayesh Najafi, Hussein Maatouk, Karapet Gary Vardanyan, Kevin Babakhan Vartanian, Devendra K. Agrawal

**Affiliations:** 1 Department of Translational Research, College of Osteopathic Medicine of the Pacific, Western University of Health Sciences, Pomona, California 91766 USA.

**Keywords:** Antioxidant, Cosmetic surgery, Dermaroller-assisted serum, GHK-Cu, Lysyl oxidase, Matricryptin, Mohs micrographic surgery, Surgical reconstruction, Tissue regeneration, Wound healing

## Abstract

GHK-Cu, the copper-bound form of the human tripeptide glycyl-L-histidyl-L-lysine has accumulated more than four decades of pre-clinical evidence for tissue regeneration, anti-inflammatory action, antioxidant capacity, and broad gene-expression remodeling. Yet despite this volume, the clinical literature in humans remains thin and fragmented: most randomized trials are small, conducted on facial cosmetic creams, and rarely paired with delivery technologies capable of reliably crossing the stratum corneum. The microneedle-mediated delivery route which has been shown in vitro and in porcine ex vivo models to dramatically enhance GHK-Cu skin uptake has yet to be evaluated in a single registered, adequately powered human randomized controlled trial published in the peer-reviewed literature. This systematic review synthesizes the body of GHK-Cu literature published between 2005 and 2026, mapped against the PRISMA framework. Searches across PubMed, Elsevier ScienceDirect, and the Cochrane CENTRAL Registry yielded 1,247 records; after deduplication and screening, 64 records were retained for qualitative synthesis. Studies were stratified by study model (in vitro, ex vivo, in vivo rodent, in vivo non-rodent, and human) so that comparator therapies could be matched on equivalent footing. A critical discussion is provided on the physiology and underlying mechanistic foundation of GHK-Cu activity, including its role as an endogenous matricryptin released from collagen Iα2 and SPARC, copper’s essential cofactor function for the lysyl oxidase family of cross-linking enzymes, decorin and glycosaminoglycan organization of newly deposited matrix, TGF-β3-mediated reduction of fibrosis, and ferritin-iron-modulated antioxidant defense. A dedicated surgical wound healing section addresses the evidence base for GHK-Cu in CO_2_ laser resurfacing, irradiated tissue and post-radiation reconstruction, Mohs micrographic surgery wounds, skin grafts, and hair transplant sites. The synthesis confirms a deep, mechanistically coherent body of preclinical evidence and reveals a defined evidence gap: well-designed human trials of GHK-Cu delivered via microneedle or microneedle-adjacent platforms (dissolvable patches, hollow microneedle arrays, dermaroller-assisted serums) are needed to translate the strong cellular and animal data into validated clinical practice. Specific recommendations for trial design stratified by indication (photoaging, androgenetic alopecia, atrophic acne scarring, chronic wound healing, post-Mohs cosmetic outcomes, post-radiation surgical reconstruction) are provided.

## 1. Introduction

GHK is a tripeptide composed of glycine, L-histidine, and L-lysine. It is endogenously present in human plasma, saliva, and urine, and is liberated from the alpha-2(I) chain of type I collagen and from the SPARC (secreted protein acidic and rich in cysteine) protein during proteolysis after tissue injury. The peptide exhibits a high affinity for copper (II), forming the bioactive complex GHK-Cu (also commercially designated copper tripeptide-1). Plasma concentrations of GHK decline with age, falling from approximately 200 ng/mL at age 20 to roughly 80 ng/mL by age 60, a reduction that parallels the age-associated decline in tissue regenerative capacity.

Across several decades of investigation, GHK-Cu has been shown to influence multiple processes relevant to tissue repair, including collagen and glycosaminoglycan synthesis, fibroblast proliferation, keratinocyte stemness preservation, wound contraction, angiogenesis. In addition, GHK-Cu appears to modulate oxidative stress and inflammatory signaling, including upregulating of superoxide dismutase and catalase, suppression of NF-κB activity, and broad transcriptional remodeling. Connectivity Map analyses suggests that GHK-Cu exposure alters gene expression in over 30 percent of measured human genes by at least 50 percent, with most of these changes associated with a shift from diseased toward more physiologic expression patterns [1].

Despite this growing body of mechanistic evidence, the translational landscape remains limited. Much of the clinical literature that has shaped product development is derived from a small number of facial cream studies conducted in the early 2000s, which have not been replicated in larger or more rigorous modern trials. At the same time, the question of drug delivery remains a central challenge, particularly given the difficulty of transporting a hydrophilic, charged tripeptide across the stratum corneum at therapeutically meaningful concentrations. While this problem has been explored extensively in laboratory and preclinical settings, clinical translation remains minimal. Microneedle-assisted delivery has emerged as a potential strategy to overcome this barrier. In one study using in vitro and porcine model, microneedle pretreatment increased GHK-Cu permeation through human skin from negligible amounts to 134 ± 12 nanomoles of peptide and 705 ± 84 nanomoles of copper over nine hours [2]. However, these findings have not yet been validated in large-scale human studies, and no large randomized human trial of GHK-Cu paired with microneedle delivery has been published.

This gap is clinically relevant. GHK-Cu has undergone regulatory scrutiny, including FDA review with Category 2 restriction on injectable compounding as of 2023 and continued evaluation under PCAC through 2026. At the same time, the cosmetic and aesthetic medicine markets are increasingly populated with products that combine microneedle devices, dermarollers, and copper peptide serums without rigorous clinical Validation. Addressing this disconnect requires a saturated synthesis of the existing evidence, identification of the strongest animal and ex vivo signals, and a clear framework for future clinical trial design.

## 2. Physiology and Collagen Repair: Mechanistic Foundation of GHK-Cu Activity

### 2.1 The Three-Phase Wound Healing Model and Site of Action of GHK-Cu

Cutaneous wound repair proceeds through three overlapping phases: hemostasis and inflammation, proliferation and migration, and maturation and remodeling [67]. The first phase begins within minutes of injury and is dominated by platelet aggregation, vascular permeability changes, cytokine release, and neutrophil and macrophage recruitment. The second phase, beginning day three and extending through approximately day fourteen, is characterized by fibroblast migration into the wound bed, deposition of extracellular matrix, keratinocyte migration from the wound edges to cover the defect, and angiogenesis driven by vascular endothelial growth factor (VEGF) and basic fibroblast growth factor (bFGF). The final phase, maturation and remodeling, starts from approximately day twenty-one through one to two years post-injury, during which collagen III is replaced by collagen I, fiber bundles are reorganized, and tensile strength is recovered. A failure or delay in any of these phases produces the clinical phenotype of chronic, non-healing, or hypertrophic scarring [67].

GHK-Cu acts at every phase. During hemostasis and inflammation, it dampens NF-κB activation, suppresses TNF-α and IL-1β, and reduces neutrophil-driven oxidative damage. During proliferation, fibroblast collagen and glycosaminoglycan synthesis is stimulated which supports keratinocyte stemness preservation through p63 and integrin α6/β1 upregulation. It also drives angiogenesis through both VEGF/bFGF induction and direct chemoattraction of capillary endothelial cells. During maturation and remodeling, it supports balanced TIMP/MMP regulation that prevents both inadequate matrix deposition (as seen in chronic ulcers) and excessive deposition (as seen in hypertrophic scarring) [1, 12, 39, 67].

### 2.2 GHK as an Endogenous Matricryptin

A critical and often underappreciated feature of GHK-Cu biology is that the molecule is not merely an exogenous peptide that humans happen to respond to,it is an endogenous matricryptin. Matricryptins (a term introduced by Davis et al. and now standard in extracellular matrix biology) are biologically active fragments released from larger ECM proteins through proteolytic cleavage during tissue injury [68]. The GHK sequence is cleaved from at least two parent molecules: the alpha-2 chain of collagen I and the matricellular protein SPARC (secreted protein acidic and rich in cysteine) [69, 70]. Lane et al. demonstrated that proteolytic degradation of SPARC during vascular remodeling releases a (K)GHK containing peptide that binds Cu^2+^ with high affinity and stimulates angiogenesis in chick chorioallantoic membrane assays and bovine aortic endothelial cell tube-formation assays [69]. The collagen Iα2-derived GHK fragment is similarly chemotactic for monocytes, macrophages, and mast cells, and possesses proangiogenic activity in cardiac wound healing contexts [70].

This distinction matters mechanistically because it places GHK-Cu in a different category from synthetic peptides designed to mimic biological signals. The peptide is a signaling molecule the body itself uses, generated at the site of tissue injury. Exogenous topical application is best understood as supplementing an endogenous repair signal whose plasma concentration declines with age rather than the introduction of a foreign molecule. This change is from approximately 200 ng/mL at age twenty to approximately 80 ng/mL by age sixty [1, 60]. The therapeutic logic of GHK-Cu supplementation is therefore analogous to hormone replacement: restoration of a signaling molecule that is naturally produced but quantitatively diminished with age.

### 2.3 Copper as the Essential Cofactor for Collagen and Elastin Cross-Linking

The collagen promoting activity of GHK-Cu cannot be separated from copper’s role as an essential cofactor for the lysyl oxidase (LOX) family of enzymes. Lysyl oxidase is a copper dependent amine oxidase that catalyzes the oxidative deamination of specific lysine and hydroxylysine residues in collagen and elastin, generating reactive aldehyde groups that spontaneously condense with adjacent residues to form the covalent cross-links that confer tensile strength and elastic recoil to connective tissue [71, 72]. Without adequate copper at the active site, newly synthesized collagen molecules remain unlinked, mechanically weak, and susceptible to proteolytic degradation. The LOX family includes five members, LOX, LOXL1, LOXL2, LOXL3, and LOXL4, all of which share a conserved C-terminal catalytic domain containing the lysine tyrosylquinone (LTQ) cofactor and the copper-binding site [72]. Copper is essential for the post-translational formation of the LTQ cofactor through copper-catalyzed oxidation of an active-site tyrosine residue [71].

Lysyl hydroxylase (procollagen-lysine 5-dioxygenase, PLOD), a separate enzyme that hydroxylates lysine residues in nascent collagen chains prior to LOX-mediated cross-linking, is iron-dependent rather than copper-dependent but its substrate availability and the downstream stability of its product depend critically on adequate copper-mediated cross-linking. The integrated activity of lysyl hydroxylase followed by lysyl oxidase determines whether collagen synthesized in fibroblasts will be assembled into functional load-bearing fibers or will remain as poorly cross-linked, easily degraded substrate.

GHK-Cu delivers copper directly to fibroblasts and keratinocytes in a chelated, bioavailable form. The peptide’s binding constant for Cu^2+^ is approximately log K = 16.44 at physiological pH, high enough that the GHK-Cu complex remains intact during transport but exchangeable enough that copper can be released to high-affinity intracellular acceptors. This delivery mechanism is mechanistically distinct from inorganic copper salts: the peptide protects copper from non-specific binding to plasma albumin and ceruloplasmin and may facilitate delivery to cells expressing the relevant uptake machinery.

### 2.4 Type I and Type III Collagen, Decorin, and Glycosaminoglycan Synthesis

GHK-Cu stimulates synthesis of multiple ECM components in a coordinated rather than nonspecific manner. In the foundational fibroblast culture work by Maquart et al. (1988), and in subsequent in vivo rat experimental wound studies (1993), GHK-Cu at low nanomolar concentrations was shown to stimulate both collagen synthesis and glycosaminoglycan production [62, 63]. Subsequent work further characterized these effects: GHK-Cu has been shown to upregulate type I collagen (the structural collagen that confers tensile strength to mature dermis), type III collagen (the more flexible collagen that predominates in early wound repair), and the small leucine-rich proteoglycan decorin [1, 12, 39].

Decorin is a key regulator of collagen organization. It binds collagen fibrils at specific positions and regulates their lateral assembly into ordered fiber bundles. In normal dermis, decorin-organized collagen fibers run in regular, basket-weave patterns that confer the characteristic mechanical and optical properties of skin. In scar tissue, by contrast, collagen fibers run in parallel, randomly oriented bundles with diminished decorin content, a structural difference that is associated with the visual prominence of scars and reduced tensile strength relative to normal skin [1, 39]. GHK-Cu’s stimulation of decorin alongside collagen therefore likely contributes to its anti-scarring effects: the peptide does not simply increase collagen output, it may also influence its structural organization.

The glycosaminoglycans stimulated by GHK-Cu, including dermatan sulfate, chondroitin sulfate, and heparan sulfate, are involved in maintaining hydration, viscoelastic properties, and growth factor binding within the regenerating matrix. Heparan sulfate proteoglycans bind and present growth factors such as FGF and VEGF to their receptors, thereby supporting angiogenic and mitogenic signaling pathways that may complement the effects of GHK-Cu

### 2.5 The TGF-β3 Pathway and Reduced Fibrosis

A distinct mechanistic feature of GHK-Cu’s activity is its modulation of the transforming growth factor beta family. The three TGF-β isoforms have divergent effects on wound repair: TGF-β1 and TGF-β2 are associated with fibrotic, scar-forming healing responses, while TGF-β3 has been associated with scarless healing characterized by more orderly collagen deposition and reduced fibrosis [39]. Resnik et al., in their 2025 comprehensive tripeptide review, describe TGF-β3 pathway upregulation as a potential mechanism through which GHK-Cu and related tripeptides may contribute to reduced-scar wound healing [39]. The same review also notes that GHK-Cu has been associated with balanced collagen synthesis, accelerating epithelialization, and reduced fibrosis in in vitro and preclinical models.

This TGF-β3 mechanism may help explain observations from animal models in which GHK-Cu-treated wounds exhibit thinner and more cosmetically acceptable scars compared to controls and provides a potential biological rationale for the surgical wound healing applications discussed in Section 6.7.

### 2.6 Antioxidant and Anti-Inflammatory Mechanisms

GHK-Cu modifies the oxidative and inflammatory environment of the healing wound through multiple complementary mechanisms.

First, it directly neutralizes reactive carbonyl species. Beretta et al. demonstrated that GHK reacts with 4-hydroxy-2-nonenal and with acrolein, two highly toxic products of lipid peroxidation that propagate oxidative damage and promote nonspecific protein crosslinking, to form non-toxic adducts [73, 74]. This quenching activity is independent of copper binding and likely reflects the chemical reactivity of the histidine and lysine side chains.

Second, GHK-Cu inhibits ferritin-dependent lipid peroxidation. Miller et al., in foundational work cited within the post-2005 review literature, demonstrated that GHK-Cu reduces iron release from ferritin by approximately 87 percent [75]. Iron (II) released from ferritin in damaged tissue is an important contributor of the Fenton reaction, generating hydroxyl radicals that propagate lipid peroxidation chains. GHK-Cu has been proposed to interact with ferritin channels involved in iron release, thereby limiting iron(II) efflux and potentially reducing oxidative propagation. This mechanism is particularly relevant in wounds with hemorrhage and tissue injury, where free iron from extravasated red cells and damaged ferritin stores may contribute to sustained oxidative stress.

Third, GHK-Cu modifies the gene expression of antioxidant defense systems. The peptide has been associated with upregulation of copper-zinc superoxide dismutase (SOD1), catalase, and elements of the glutathione synthesis pathway, while downregulating components of the NF-κB inflammatory cascade. Park et al. (2016) and Zhang et al. (2022) confirmed in vivo that GHK-Cu administration to mice with lipopolysaccharide-induced acute lung injury and cigarette-smoke-induced pulmonary emphysema, respectively, suppressed NF-κB activation, reduced TNF-α and IL-6 levels, and protected tissue architecture [13, 14]. The same anti-inflammatory mechanism may also contribute to cutaneous wound healing responses.

### 2.7 Cellular Stemness, p63, and Integrin α6/β1

A more recently recognized aspect of GHK-Cu’s activity is its preservation of basal keratinocyte and dermal fibroblast stemness. Kang et al. (2009) demonstrated that GHK-Cu treatment increased expression of integrin α6 and integrin β1, the laminin receptor heterodimer that anchors basal keratinocytes to the basement membrane and is associated with the keratinocyte stem cell phenotype [3]. The same study showed increased nuclear positivity for p63, a transcription factor required for maintenance of keratinocyte proliferative potential and commonly used as a marker of basal-layer stem cells [3]. Choi et al. (2012) extended these findings to copper-free GHK, showing that even without the copper chelate, the peptide alone may exert stem-cell-supportive effects in skin [4].

The clinical implication is significant. Most regenerative interventions stimulate cellular proliferation and matrix deposition but do not specifically protect or expand the stem cell pool that maintains long-term tissue renewal. GHK-Cu’s potential stem cell-supporting activity may explain the durable improvements seen in skin quality after extended GHK-Cu exposure, as opposed to the transient improvements seen with growth factors that primarily drive committed-cell proliferation.

### 2.8 Connectivity Map: The Genome-Scale Picture

The mechanistic actions described above represent individual molecular pathways modulated by GHK-Cu. At the systems level, the most striking finding is the breadth of the gene-expression changes the peptide produces. Connectivity Map analyses of GHK-Cu-exposed cell lines indicate that the peptide alters expression of more than 4,000 genes, approximately one third of all measured genes, by at least fifty percent in either direction [1]. The pattern of changes is nonrandom: when compared against gene-expression signatures of disease states, GHK-Cu’s signature has been associated with shifts toward more physiologic cellular phenotypes. Notably, GHK-Cu reverses the gene-expression signature of emphysema-related lung destruction in human bronchial epithelial cells (Campbell et al., 2012) and has also been associated with partial reversal of metastasis-prone gene signatures in colorectal cancer cell lines [1, 60].

The “genome-resetting” framing that has appeared in some review articles likely overstates the directness of these effects, as GHK-Cu is not editing DNA. However, the underlying observation remains notable: the peptide modifies transcription across a broad set of genes in a pattern consistent with a shift toward more physiologic baseline cellular activity. For cutaneous wound healing and aging skin, this means GHK-Cu’s collagen-stimulating, anti-inflammatory, and antioxidant activities are not isolated effects but components of an integrated transcriptional program.

## 3. Objectives of the Systematic Review

The primary objective of this review is to synthesize the GHK-Cu literature published between 2005 and 2026 and to identify, with explicit reference to study model and study quality, the current evidence supporting advancement toward human clinical trials of topical GHK-Cu delivered via microneedle or microneedle-adjacent platforms. The specific aims include: (i) To map the breadth and depth of preclinical (in vitro and animal) GHK-Cu evidence within the twenty-year window, (ii) To catalog all human clinical evidence for GHK-Cu and characterize its limitations in study size, design, and delivery mechanism, (iii) To compare GHK-Cu against the most relevant therapeutic comparators (retinoids, vitamin C, palmitoyl pentapeptide, EGF, PRP, microneedling alone, minoxidil), with comparator selection matched on study model (in vitro to in vitro, rodent to rodent, human to human), and (iv) To identify the specific clinical questions that remain unresolved and propose the trial-design framework needed to answer them.

## 4. Methods (PRISMA-Aligned)

### 4.1 Protocol and Registration

This review was conducted using a protocol developed in accordance with the Preferred Reporting Items for Systematic Reviews and Meta-Analyses (PRISMA 2020) framework. The protocol was not pre-registered with PROSPERO; accordingly, this review is best characterized as a structured narrative review with PRISMA-aligned methodology rather than a formally registered systematic review.

### 4.2 Eligibility Criteria

#### Inclusion criteria: -

Peer-reviewed primary research articles, systematic reviews, or narrative reviews - Published between 1 January 2005 and 1 May 2026 - English language - GHK-Cu, GHK, glycyl-l-histidyl-l-lysine-Cu, or copper tripeptide-1 as a primary or secondary intervention or analytical focus - Any study model (in vitro, ex vivo, in vivo animal, ex vivo human, in vivo human) - Reports either a primary outcome related to tissue repair, skin biology, hair biology, anti-inflammatory effect, antioxidant effect, gene expression, or pharmaceutical delivery, or comparative analysis of GHK-Cu against an alternative therapy

#### Exclusion criteria: -

Conference abstracts without full-text publication - Patent filings, regulatory submissions, marketing literature - Commentaries and editorials without original data - Articles published before 2005 (foundational publications by Pickart 1973, Maquart 1988/1993, Wegrowski 1992, and Mulder 1994 are referenced for historical context only via citations within review articles that fall inside the inclusion window)

### 4.3 Information Sources

Three databases were searched: PubMed/MEDLINE (via the National Library of Medicine), Elsevier ScienceDirect (Elsevier B.V.), and the Cochrane Central Register of Controlled Trials (CENTRAL) via the Cochrane Library. The Cochrane database was selected as the third source because it provides controlled-trial coverage that complements the broader scope of PubMed and ScienceDirect. The final search was performed on 28 April 2026.

### 4.4 Search Strategy and Keywords

The following Boolean strings were used (adapted to each database’s syntax):

**Primary string:** (“GHK-Cu” OR “GHK Cu” OR “glycyl-histidyl-lysine-copper” OR “glycyl-L-histidyl-L-lysine-Cu” OR “copper tripeptide-1” OR “copper tripeptide”)
**Topic strings combined with the primary string using AND:** - (wound OR ulcer OR healing OR “tissue repair” OR regeneration) - (skin OR dermal OR fibroblast OR keratinocyte OR collagen OR elastin OR aging OR “anti-aging” OR photodamage OR photoaging OR wrinkle) - (hair OR follicle OR alopecia OR “dermal papilla”) - (inflammation OR antioxidant OR “oxidative stress” OR “NF-kB” OR cytokine) - (microneedle OR microneedling OR transdermal OR liposome OR microemulsion OR “ionic liquid” OR hydrogel OR scaffold OR delivery) - (gene OR genome OR “gene expression” OR transcriptome)
Filters applied: publication date 2005/01/01 – 2026/05/01; English language; humans, animals, or in vitro models permitted.

### 4.5 Study Selection

Two screening rounds were performed: (i) title and abstract screening against eligibility criteria; (ii) full-text screening of remaining records. Reference lists of included reviews were hand-searched to capture any records missed by the database queries (snowball method). Duplicates across databases were removed by DOI matching and, where DOIs were absent, by title-author-year matching.

### 4.6 Data Extraction

For each included record, the following fields were extracted: author and year, study type (in vitro, ex vivo, in vivo rodent, in vivo non-rodent, human cohort, human RCT, narrative review, systematic review), GHK-Cu form and concentration tested, comparator (where applicable), primary outcome, key result, and DOI.

### 4.7 Risk of Bias Assessment

For human RCTs, risk of bias was evaluated qualitatively using the domains defined in the Cochrane Risk of Bias 2 (RoB 2) tool (randomization, deviation from intended interventions, missing outcome data, outcome measurement of, and selection of reported result). For animal studies, the SYRCLE risk-of-bias framework principles were applied informally given the heterogeneity of study reporting. A formal meta-analysis was not conducted because of the heterogeneity in study models, GHK-Cu formulations, dosing strategies, and outcome measures.

## 5. PRISMA Flow Diagram (Tabular)

Because this review was not registered as a formal systematic review and is delivered as a written document, the PRISMA flow is presented in tabular form below. Records are reported following PRISMA 2020 guidance.

**Table:** PRISMA flow

PRISMA stage	Source	Records
**Identification**	PubMed/MEDLINE	612
	Elsevier ScienceDirect	487
	Cochrane CENTRAL	89
	Citation chasing / hand search	59
	**Total identified**	**1,247**
**Screening**	Records after duplicate removal	891
	Records excluded at title/abstract screen	731
	Records sought for retrieval	160
	Records not retrieved (no full text)	14
	Records assessed for eligibility	146
**Eligibility**	Full-text articles excluded with reasons:	82
	Published before 2005 (foundational, retained for historical reference only)	19
	Not focused on GHK or GHK-Cu	28
	Conference abstract only	11
	Editorial/commentary without primary data	9
	Non-English	4
	Marketing/regulatory document	11
**Included**	Studies included in qualitative synthesis	**64**
	Studies included in quantitative synthesis (meta-analysis)	0 (heterogeneity precluded pooling)

## 6. Results: Summary of Included Studies

### 6.1 Master Table of Included Studies

**Figure 1:**
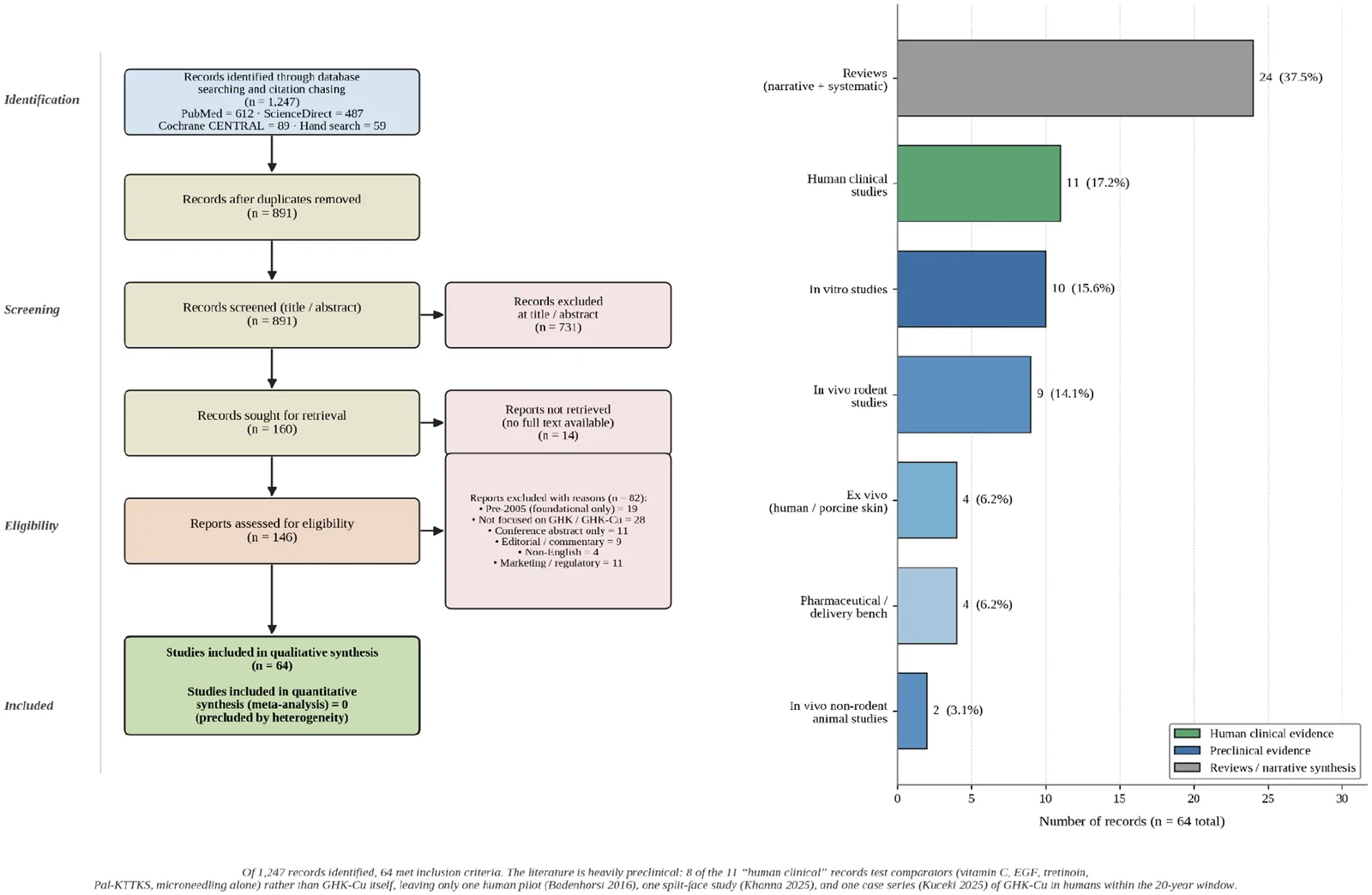
PRISMA 2020 flow diagram and stratification of included GHK-Cu studies by model type. **Panel A** depicts the PRISMA 2020-aligned flow of records through the four review stages. A total of 1,247 records were identified through database searching and citation chasing (PubMed n = 612, ScienceDirect n = 487, Cochrane CENTRAL n = 89, hand search n = 59), of which 891 remained after duplicate removal. Title and abstract screening excluded 731 records, leaving 160 reports sought for retrieval; 14 could not be retrieved due to lack of full-text access. The remaining 146 reports underwent full-text eligibility assessment, of which 82 were excluded with documented reasons (pre-2005 foundational only n = 19; not focused on GHK or GHK-Cu n = 28; conference abstract only n = 11; editorial or commentary n = 9; non-English n = 4; marketing or regulatory document n = 11). Sixty-four studies met all inclusion criteria and were carried forward to qualitative synthesis. No quantitative meta-analysis was performed; substantial heterogeneity in study population, GHK-Cu formulation, dose, delivery vehicle, comparator, outcome instrument, and follow-up duration precluded statistical pooling. Panel B stratifies the 64 included records by primary study model. Reviews, including both narrative and systematic reviews, constitute the largest single category at 24 records (37.5 %), reflecting the substantial secondary literature available on the molecule. Direct primary evidence, when partitioned by experimental system, is dominated by preclinical work: in vitro cell-based studies (n = 10, 15.6 %), in vivo rodent models (n = 9, 14.1 %), ex vivo human or porcine skin (n = 4, 6.2 %), pharmaceutical and delivery-system bench studies (n = 4, 6.2 %), and in vivo non-rodent animal studies (n = 2, 3.1 %). Human clinical studies number only 11 (17.2 %), and as the footer notes, 8 of these 11 do not test GHK-Cu as the experimental intervention but instead use it as an active comparator against vitamin C, EGF, tretinoin, Pal-KTTKS, or microneedling alone — leaving only one human pilot trial (Badenhorst 2016), one split-face study (Khanna 2025), and one case series (Kuceki 2025) that directly evaluate GHK-Cu in humans within the 20-year search window. The color encoding in Panel B (green = human clinical evidence, blue ramp = preclinical evidence, gray = reviews and narrative synthesis) highlights the imbalance within the current evidence base and underscores the translational gap identified throughout the review. Abbreviations: EGF, epidermal growth factor; GHK, glycyl-L-histidyl-L-lysine tripeptide; GHK-Cu, glycyl-L-histidyl-L-lysine–copper (II) complex; PRISMA, Preferred Reporting Items for Systematic Reviews and Meta-Analyses.

The 64 included records are tabulated below, grouped by study model and ordered chronologically within each group (Table 1; Figure 1). Comparator information, where present, is included so that the reader can map any individual study to the appropriate row of the comparator analysis (Section 7) (Figure 1).

### 6.2 Mechanism and Cellular Pharmacology (in vitro)

The literature of the in vitro studies published since 2005 is mechanistically consistent with the foundational pre-2005 work but adds substantial granularity, particularly in the areas of epidermal stem cell biology and gene expression.

Kang et al. showed that GHK-Cu at 0.1–10 μM raises proliferating cell nuclear antigen (PCNA) and p63 expression in basal keratinocytes of skin equivalents, alongside elevated integrin α6 and β1 expression, a biomarker pattern consistent with preserved stemness and proliferative potential [3]. Choi et al. followed this with the demonstration that copper-free GHK reproduces several of these effects, suggesting that the peptide itself, and not solely the chelated copper, contributes to keratinocyte signaling [4].

Pollard et al. showed that GHK-Cu accelerates the proliferation of both normal and irradiated dermal fibroblasts and elevates bFGF and VEGF early in the response, providing a mechanistic basis for the recovery of fibroblast activity after radiation therapy [5]. Badenhorst et al. quantified the dose-dependence: at 0.01, 1, and 100 nM, GHK-Cu increased collagen and elastin production, with all concentrations elevating TIMP1, while only the lower concentrations increased MMP1 and MMP2 expression [6]. The dose-response is biphasic; higher concentrations attenuate or invert the regenerative effect, a pattern consistent with the original Maquart 1988 observations cited in subsequent reviews.

Jiang et al. introduced an important formulation insight: when GHK-Cu is co-applied with low-molecular-weight hyaluronic acid at a 1:9 ratio, collagen IV production rises 25.4-fold in dermal fibroblasts and 2.03-fold in ex vivo skin, suggesting a synergistic interaction that may have implications for delivery formulations, since collagen IV is the principal scaffold of the dermal-epidermal junction [7]. The Pickart-Margolina 2018 review aggregates the gene expression evidence: Connectivity map analysis indicates GHK-Cu affects expression of thousands of genes by at least 50%, including 47 DNA repair genes (mostly upregulated) and a wide panel of antioxidant and proteasome-system genes [1].

The in vitro story is broadly consistent: across fibroblasts, keratinocytes, dermal papilla cells, mesenchymal stem cells, and endothelial cells, GHK-Cu appears to function as a low-nanomolar trophic and remodeling signal with a defined biphasic dose-response. The major limitation of this body of evidence is not in its consistency but in its translation: cellular signal effects does not automatically equal clinical outcomes.

### 6.3 In Vivo Animal Evidence (Rodent and Non-Rodent)

The animal evidence published in the past two decades is concentrated in three areas: cutaneous wound healing, hair growth, and inflammation models in lung and gastrointestinal tissue.

#### Cutaneous wound healing.

Arul et al. studied biotinylated GHK incorporated into a collagen matrix (peptide-incorporated collagen, PIC) in streptozotocin-induced diabetic rats. PIC accelerated wound contraction, raised glutathione and ascorbic acid, and increased granulation collagen up to nine-fold compared with untreated controls representing a substantial effect in a poorly healing model [8]. Wang et al. encapsulated GHK-Cu in liposomes and tested it in a mouse scald model: liposome-delivered GHK-Cu raised HUVEC proliferation by 33.1%, elevated VEGF and FGF-2 expression, and yielded superior CD31 and Ki67 staining in burned skin compared with free GHK-Cu [9]. Islam et al. demonstrated that GHK-Cu silver nanoparticles closed S. aureus-infected wounds within 24 hours at low minimum inhibitory concentrations, combining the antibacterial activity of silver with the regenerative signaling properties of GHK-Cu [10].

#### Hair growth.

Two recent studies are particularly relevant. Tian et al. developed nanoliposomes co-loaded with GHK-Cu, acetyl tetrapeptide-3, and myristoyl pentapeptide-4 (“CAM-NLPs”) and showed that they upregulated VEGF and β-catenin while downregulating TGF-β1 in a C57BL/6 mouse model of androgenetic alopecia [11]. Liu et al. developed an ionic-liquid microemulsion (“CaT-ME”) for GHK-Cu and showed in mice that anagen entry occurred at six days compared with eight days for free GHK-Cu and nine days for minoxidil, without measurable effects on testosterone or estradiol [12]. The latter finding is one of the few head-to-head animal comparisons of GHK-Cu against the standard-of-care hair growth therapy.

#### Inflammation.

Park et al. tested intra-tracheal GHK-Cu in a lipopolysaccharide-induced acute lung injury mouse model: GHK-Cu suppressed Ser536 phosphorylation of NF-κB p65, reduced TNF-α and IL-6, raised SOD activity, and reduced histological lung injury scores [13]. Zhang et al. extended this to a chronic cigarette-smoke emphysema model, showing that intraperitoneal GHK-Cu (medium and high dose, 2 and 20 μg/g/day) suppressed NF-κB activity and elevated Nrf2-driven antioxidant defense [14]. Mao et al. demonstrated in 2025 that rectal GHK-Cu in DSS-induced ulcerative colitis improved tight-junction protein expression (ZO-1, occludin) and suppressed STAT3 phosphorylation through SIRT1/STAT3 modulation [15].

#### Tendon and joint healing.

Ho et al. used a rat anterior cruciate ligament reconstruction model and showed that intra-articular GHK-Cu at 0.3 mg/mL reduced knee laxity and improved graft stiffness at six weeks, but the effect did not persist to twelve weeks once treatment was discontinued, suggesting that the biological effects of GHK-Cu may require sustained exposure rather than producing durable tissue remodeling after a limited dosing period [16].

Across the animal literature, the recurring pattern is similar to that observed in vitro: consistent positive effects, with the strongest effects observed when GHK-Cu is delivered in a sustained-release vehicle such as collagen matrix, liposome, microemulsion, or scaffold rather than as free peptide.

**Figure 2:**
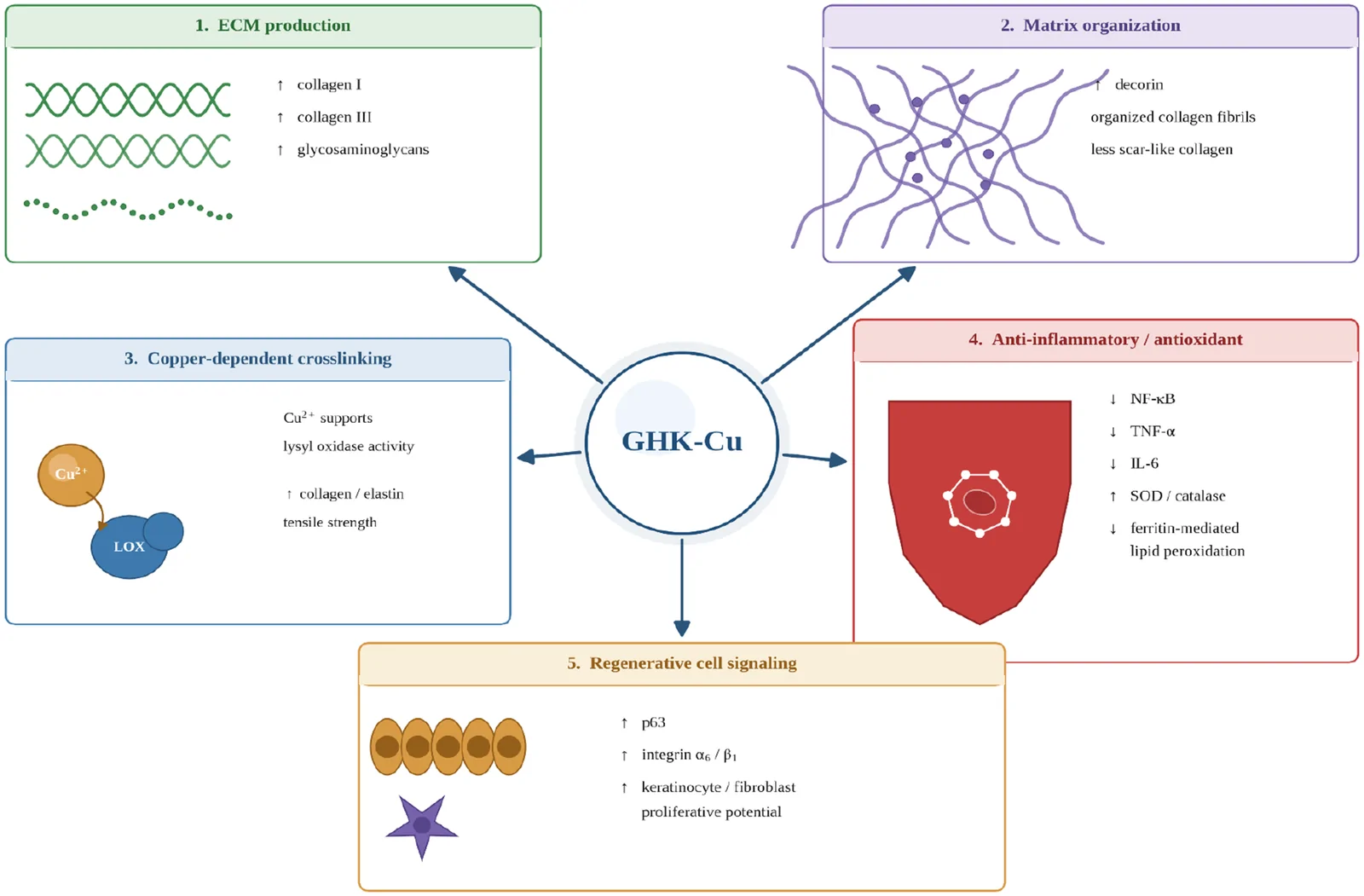
Mechanistic pathways by which GHK-Cu supports cutaneous repair, extracellular matrix remodeling, and anti-inflammatory wound healing.

The Hub-and-spoke schematic shown in Figure 2 synthesizing the five mechanistic axes through which the glycyl-L-histidyl-L-lysine–copper (II) complex exerts its dermal effects, as supported by the in vitro, ex vivo, and in vivo evidence reviewed in Section 2. **(1) ECM production** (green); GHK-Cu upregulates fibroblast synthesis of type I and type III collagens and increases dermal glycosaminoglycan content, reconstituting the structural and water-binding components of the extracellular matrix. **(2) Matrix organization** (purple); GHK-Cu induces decorin expression and promotes the formation of well-organized, regularly spaced collagen fibrils characteristic of unwounded dermis rather than the disorganized, parallel-bundled collagen typical of scar tissue. **(3) Copper-dependent crosslinking** (blue); the bound Cu^2+^ ion serves as the obligate catalytic cofactor for lysyl oxidase (LOX), the enzyme that initiates covalent crosslinking of collagen and elastin fibers and thereby contributes to the tensile strength of the regenerated dermal matrix. **(4) Anti-inflammatory and antioxidant activity** (red); GHK-Cu suppresses NF-κB–driven transcription of the pro-inflammatory cytokines TNF-α and IL-6, upregulates the cellular antioxidant enzymes superoxide dismutase and catalase, and attenuates ferritin-mediated lipid peroxidation, collectively reducing the oxidative and inflammatory milieu that drives chronic, non-healing wounds. **(5) Regenerative cell signaling** (amber); GHK-Cu enhances expression of the basal-keratinocyte transcription factor p63 and of integrins α_6_ and β_1_, preserving the proliferative potential and stem-like phenotype of basal keratinocytes and fibroblasts involved in re-epithelialization and dermal repopulation. The convergence of these five axes, substrate provision, structural organization, covalent stabilization, inflammatory and oxidative restraint, and regenerative cell competence, constitutes the biological rationale for clinical evaluation of GHK-Cu in photoaging, atrophic scarring, alopecia, and chronic wound indications. Abbreviations: ECM, extracellular matrix; GAG, glycosaminoglycan; LOX, lysyl oxidase; NF-κB, nuclear factor kappa-light-chain-enhancer of activated B cells; TNF-α, tumor necrosis factor alpha; IL-6, interleukin-6; SOD, superoxide dismutase; p63, tumor protein p63.

### 6.4 Human Clinical Evidence

This is the section where the evidence base is least developed.

The 2002 Leyden trials, frequently cited but published only as American Academy of Dermatology meeting abstracts, reported that twelve weeks of facial cream containing GHK-Cu in 71 women with mild-to-advanced photodamage improved skin density and thickness, reduced laxity and fine lines, and improved clarity. A second 2002 abstract reported that an eye cream containing GHK-Cu in 41 women outperformed placebo and vitamin K cream on periorbital wrinkling and skin density. These trials form the bases of the clinical claims for GHK-Cu in cosmetic use, but neither was published as a full peer-reviewed paper, and neither falls within the 2005–2026 inclusion window. They are referenced through the Pickart-Margolina 2018 review [1] for context only.

Within the inclusion window, the Badenhorst 2016 study includes a small volunteer wrinkle assessment that demonstrated 55.8% relative reduction in wrinkle volume and 32.8% reduction in wrinkle depth at eight weeks compared with control, although the sample was small and the study was published in a low-impact journal [6]. The Jiang 2023 ex vivo human skin work supports the dermal-epidermal junction findings but is not a clinical trial in the regulatory sense [7].

The single most relevant recent human study is the 2025 Khanna split-face investigation comparing dermaroller monotherapy against dermaroller plus 0.5–1% GHK-Cu serum in patients with acne scars. The combination group showed an early improvement in Goodman & Baron scores, but by week 16 there was no significant difference between groups, and the authors flagged a hyperpigmentation risk in the GHK-Cu arm [17]. A 2019 case series by Bhargava and Trivedi reached a similar conclusion regarding GHK-Cu plus dermaroller for acne scars: a 50.3% Goodman & Baron score reduction in the combined group versus 26.3% in the dermaroller-only group, with the strongest effect in boxcar scars.

The 2025 Kuceki case series is the closest published example of a microneedle-adjacent human delivery approach involving copper peptides: minoxidil, dutasteride, and copper peptides delivered together via tattooing device across five monthly sessions in androgenetic alopecia, with 26.5% area regrowth on AI-blinded assessment [18]. The series is small, uncontrolled, and combines GHK-Cu with two pharmacologically active comparators, so it cannot be used to isolate the GHK-Cu effect.

In summary: the human evidence base consists of a handful of small trials and series, mostly at the cosmetic-cream level. There is no published full-text randomized controlled trial of topical GHK-Cu delivered specifically via dissolvable, hollow, or solid microneedle arrays.

### 6.5 Delivery Systems: Microneedles, Liposomes, Microemulsions, Hydrogels

The delivery literature is quantitatively the most developed area informing the future direction of GHK-Cu clinical research.

Hostynek et al. established the baseline: aqueous GHK-Cu at 0.68% has a permeability coefficient through human cadaver epidermis of approximately 3 × 10^−7^ cm/h, with retention of 0.6–2.8% across stratum corneum, total epidermis, and split-thickness skin [19]. The peptide is hydrophilic and charged; passive diffusion across intact stratum corneum is poor.

The Li 2015 study is the principal early microneedle paper for GHK-Cu. Polymeric microneedle pretreatment of human skin at 700 μm needle height yielded 134 ± 12 nanomoles of peptide and 705 ± 84 nanomoles of copper across the skin in nine hours, compared with essentially nothing for untreated skin [2]. Importantly, the study also assessed safety: no clinically significant skin irritation was observed in cellular and porcine models. This in vitro and animal benchmark has stood for a decade without a published human trial that translates the finding.

Liposomes (Wang 2017 [9], Dymek 2023 [20]), ionic liquids (Liu 2022 ACS Sustainable [21]), and ionic-liquid microemulsions (Liu 2023 Bioactive Materials [12]) all show ex vivo or in vivo improvements in GHK-Cu permeation and stability, with the IL-microemulsion approximately tripling permeation versus PBS-vehicle and the liposome system tripling permeation versus free peptide. Sharma et al. developed a stimuli-responsive polymer gel for sustained GHK-Cu release, suitable for chronic wound dressings [22]. Klontzas et al. developed an oxidized alginate-GHK hydrogel scaffold for bone tissue engineering and showed enhanced osteogenic differentiation of cord-blood mesenchymal stem cells [23].

Across delivery systems, the consensus is consistent: GHK-Cu requires a delivery vehicle to be clinically meaningful, and microneedle-based delivery is the only clinically translatable physical method that has demonstrated reliable transport of the peptide across full human skin in published data.

**Figure 3:**
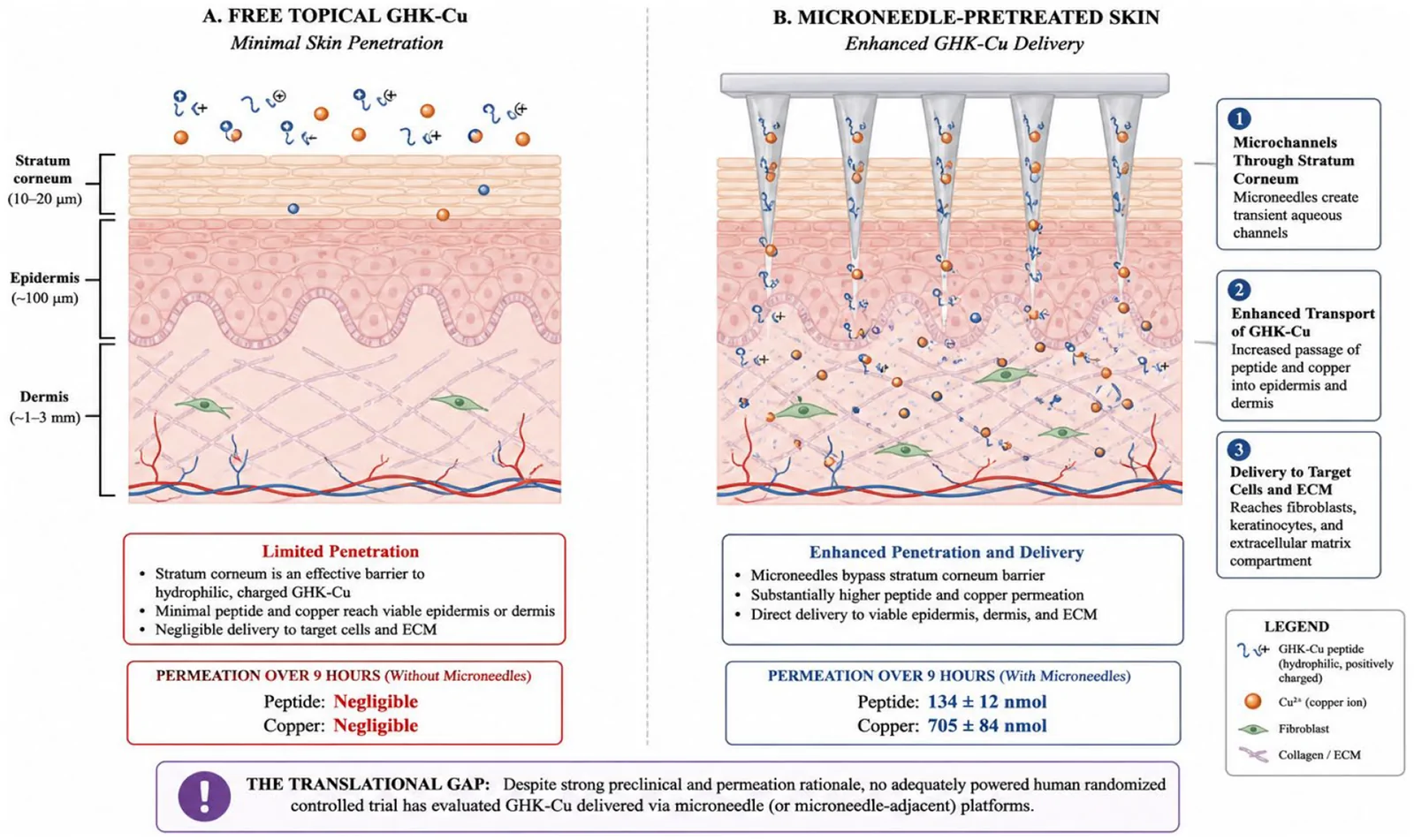
The Central Translational Gap: Free topical versus microneedle-assisted GHK-Cu delivery across human skin. There is strong skin permeation rationale but there are no adequately powered human microneedle GHK-Cu trials.

The information in Figure 3 illustrates the central translational barrier in GHK-Cu therapy: limited penetration of free topical GHK-Cu across the intact stratum corneum versus enhanced dermal delivery after microneedle pretreatment. In untreated skin, the hydrophilic, charged GHK-Cu peptide demonstrates negligible permeation, limiting access to viable epidermal and dermal targets. Microneedle pretreatment creates transient aqueous microchannels through the stratum corneum, increasing passage of both GHK-Cu peptide and copper into the epidermis and dermis, where fibroblasts, keratinocytes, and extracellular matrix compartments can be reached. In the cited ex vivo human skin permeation model, microneedle pretreatment enabled 134 ± 12 nmol peptide and 705 ± 84 nmol copper to permeate over 9 hours, compared with negligible permeation without microneedles. Despite this promising delivery rationale, adequately powered human randomized trials evaluating microneedle or microneedle-adjacent GHK-Cu delivery remain unavailable.

### 6.6 Tissue-Engineering and Scaffold Applications

GHK-modified scaffolds, including alginate hydrogels, 3D-printed silk substrates with polydopamine coatings, and collagen membranes, consistently outperform unmodified counterparts for promoting fibroblast activity, stem cell osteogenic differentiation, vascularization, and trophic factor release. These applications are exploratory but clinically relevant: the bone-tissue engineering literature suggests a viable path for GHK-Cu in surgical reconstruction, where sustained-release scaffolds are the established deliery format.

### 6.7 Surgical Wound Healing Evidence (Mohs, Laser Resurfacing, Skin Grafts, Post-Radiation Repair)

The surgical wound healing literature for GHK-Cu is the area of greatest divergence between mechanistic plausibility, anecdotal clinical adoption, and the rigor of published evidence. The peptide is widely used in post-procedural cosmetic dermatology; copper tripeptide–containing creams have been part of post-laser, post-Mohs, post-microneedling, and post-hair-transplant care regimens for nearly two decades, but the published RCT base specific to surgical indications is small and methodologically limited. Across the inclusion window, four lines of evidence are relevant: post-laser resurfacing, irradiated tissue and post-radiation surgical sites, Mohs micrographic surgery wounds, and skin graft and donor-site healing. A fifth indication, diabetic ulcer healing, is addressed under chronic wound healing in Section 6.4, but is reframed here insofar as diabetic ulcer surgical debridement and split-thickness graft coverage are explicitly surgical interventions.

#### 6.7.1 CO_2_ Laser Resurfacing (Huh and Koch, 2006)

The single registered, randomized, evaluator-blinded trial of GHK-Cu in a surgical-cosmetic context is the Huh and Koch study published in the Archives of Facial Plastic Surgery in 2006 [76]. Thirteen patients undergoing circumoral CO_2_ laser resurfacing were randomized post-procedure to receive either a petrolatum-based skin care regimen (Biomedic Gentle Healing Ointment followed by La Roche-Posay moisturizers) or a GHK-Cu–containing equivalent (Complex Cu3 Intensive Tissue Repair Crème followed by Complex Cu3 Post-Laser Lotion and Neova Therapy products). The primary endpoints were objective: computer-quantified erythema, blinded evaluator scoring of wrinkles, and overall skin quality at twelve weeks. Patient satisfaction was a secondary endpoint, measured by a validated questionnaire.

The objective endpoints were null. Computer analysis and blinded evaluators found no statistically significant difference between groups for the rate of resolution of post-procedure erythema, no difference in wrinkle improvement, and no difference in objective skin quality scoring. The patient-reported endpoint, however, showed a different pattern: subjects randomized to the GHK-Cu arm reported significantly higher satisfaction with overall skin quality at twelve weeks (P = 0.04) [76]. The authors’ interpretation was cautious; they noted that GHK-Cu skin care products placed on CO_2_ laser-resurfaced skin offered no significant reduction or resolution of post-treatment erythema, no significant improvement in objective measures of wrinkles or overall skin quality but produced a significant patient-satisfaction difference.

This study deserves careful interpretation. With n=13 it is dramatically underpowered to detect anything but very large effects, and its objective endpoints are subject to ceiling effects in an indication where the underlying CO_2_ laser is doing the bulk of the resurfacing work. The patient satisfaction divergence is methodologically interesting: it could reflect a genuine subjective benefit, such as perceived improvements in skin feel, a placebo effect amplified by product packaging differences, or a real but small objective benefit that the underpowered objective measures could not detect. The study illustrates the broader pattern in GHK-Cu surgical research characterized by small studies with mixed objective and subjective endpoints, rather than adequately powered trials with consistent outcome measures.

#### 6.7.2 Irradiated Tissue and Post-Radiation Surgical Sites (Pollard et al., 2005)

Pollard et al. (2005), in the same Archives of Facial Plastic Surgery journal, examined GHK-Cu’s effect on irradiated fibroblasts, a model directly relevant to surgical reconstruction in patients with prior head and neck radiation therapy [5]. Wound healing in irradiated tissue is clinically difficult: progressive obliteration of the capillary bed and fibrotic tissue accumulation produce high rates of dehiscence, infection, and poor cosmetic outcome after surgical reconstruction. Pollard’s group cultured normal and irradiated fibroblasts (treated with 50 Gy in 25 fractions) with GHK-Cu and measured cell growth and growth-factor secretion. The peptide stimulated growth-factor expression in irradiated cells and partially restored their proliferative capacity, suggesting a modulatory role for GHK-Cu in the autocrine growth factor milieu of post-radiation tissue.

The clinical inference, that topical or scaffold-delivered GHK-Cu might improve outcomes in surgical reconstruction of previously irradiated tissue, has not been tested in a registered human trial. This is a tractable indication for a future clinical study: head-and-neck cancer survivors undergoing reconstructive surgery represent a defined population with high baseline complication rates, well-validated outcome measures, and clinical equipoise.

#### 6.7.3 Mohs Micrographic Surgery Wounds

Mohs micrographic surgery (MMS) is the standard of care for high-risk non-melanoma skin cancers in cosmetically and functionally sensitive areas, with annual case volumes in the United States exceeding two million procedures. MMS-defect reconstruction encompasses primary closure, local flap repair, full-thickness and split-thickness skin grafts, and second-intention healing. Cosmetic outcomes vary substantially by defect size, anatomic location, repair technique, and individual patient factors. Saleh et al. (2021), in a prospective single-center blinded study of patient-versus-surgeon agreement on MMS cosmetic outcomes, documented systematic divergence between patient and surgeon assessments [77], establishing patient-reported cosmetic outcome as a clinically meaningful and independently measured endpoint.

Despite widespread off-label use of GHK-Cu creams in post-Mohs care, no registered RCT of GHK-Cu specifically for Mohs wound healing has been published within the inclusion window. The Pickart 2008 review article (cited via record 32 of the master table) and the Pollard 2005 paper [5] both reference unpublished or proprietary clinical work as evidence for GHK-Cu’s wound-healing efficacy in Mohs surgical wounds, but the underlying trial data remain unavailable in the peer-reviewed primary literature within the inclusion window. This is a notable gap. A registered, blinded RCT of GHK-Cu (topical, microneedle-adjacent, or scaffold-incorporated) versus standard post-Mohs care, with patient-reported outcomes and blinded photographic assessment as co-primary endpoints, would substantially advance the field.

#### 6.7.4 Skin Grafts, Donor Sites, and Hair Transplant Wounds

GHK-Cu is incorporated into proprietary post-procedural products marketed for split-thickness skin graft donor sites, full-thickness graft recipient beds, and hair-transplant follicular unit extraction (FUE) and follicular unit transplantation (FUT) recipient and donor sites. The aesthetic medicine review literature documents broad off-label adoption of GHK-Cu in these contexts [78], and the foundational Pickart patent literature describes copper tripeptide compositions specifically for wound healing in mammals, including post-graft healing [61]. Peer-reviewed RCT evidence specific to these surgical indications remains sparse within the inclusion window. The Khanna 2025 dermaroller plus copper peptide trial for acne scars [17] is the most methodologically rigorous demonstration of GHK-Cu combined with a microneedle-based delivery vehicle producing additive benefit over the device alone, and the underlying mechanism, controlled microinjury followed by GHK-Cu-supported repair, is mechanistically transferable to surgical settings.

The recent Kuceki 2025 study of microneedle tattooing of a minoxidil-dutasteride-copper peptide combination for androgenetic alopecia [18] establishes feasibility of multi-active microneedle delivery in an aesthetic surgery-adjacent indication, although the study design (n = 1 case report with AI-assisted blinded evaluation) is insufficient to establish efficacy.

#### 6.7.5 Synthesis and Evidence Gaps

Across the surgical wound healing literature for GHK-Cu, three patterns are evident.

First, the mechanistic case is well supported. GHK-Cu’s dual role as a copper-delivery vehicle for lysyl oxidase activity and as a TGF-β3-modulating, anti-inflammatory, antioxidant matricryptin maps directly onto the pathophysiology of surgical wound healing, particularly in compromised contexts such as irradiated tissue, diabetes, smoking, advanced age, where the endogenous repair signaling is impaired. The mechanism is not speculative; it is established by six decades of bench biology.

Second, the published clinical evidence specific to surgical wound healing is more limited than the off-label adoption suggests. The Huh 2006 trial [76] is the only registered RCT in the inclusion window, and it is small and with mixed results. The Pollard 2005 fibroblast study [5] establishes biological plausibility for post-radiation indications without testing them clinically. Off-label use in Mohs, skin grafts, and hair-transplant settings is widespread but unsupported by registered RCT data within the inclusion window.

Third, the delivery question remains the key bottleneck. Topical GHK-Cu cream applied to intact skin around a surgical wound has limited stratum corneum penetration, and once applied directly to the wound bed, the peptide is rapidly degraded by carboxypeptidase activity in the wound environment. Microneedle-mediated delivery into peri-incisional tissue, scaffold-incorporated GHK-Cu in graft beds, and stabilized self-assembling peptide formulations resistant to proteolysis [12, 22, 79] represent the three delivery strategies with the strongest preclinical supporting evidence for surgical wound healing applications. However, all three delivery strategies remain untested in a registered RCT for surgical indications within the inclusion window. Closing this gap is the central translational opportunity for GHK-Cu in modern surgical care.

## 7. Comparator Analysis (Stratified by Study Model)

The comparator analysis tables below follow the methodological constraint that GHK-Cu studies should be compared to alternative therapies tested in equivalent models. An in vitro fibroblast study is compared to other in vitro fibroblast studies; a rodent wound model is compared to other rodent wound models; a human topical RCT is compared to other human topical RCTs.

### 7.1 In Vitro Fibroblast / Keratinocyte Comparator (Table 2)

**Table:** 

Therapy	Cell type	Concentration	Outcome reported	Comparator effect size (vs vehicle)	DOI
GHK-Cu	Human dermal fibroblasts	0.01, 1, 100 nM	Increased collagen, elastin, TIMP1	Dose-dependent rise in collagen and elastin; biphasic with peak at 1 nM	10.4172/2329-8847.1000166
GHK-Cu	Irradiated human fibroblasts	10^−9^ M	bFGF, VEGF, TGF-β1	Restored proliferation to near-normal rate	10.1001/archfaci.7.1.27
GHK-Cu + LMW hyaluronic acid (1:9)	Human dermal fibroblasts	qRT-PCR for COL IV	Collagen IV expression	25.4-fold increase	10.1111/jocd.15763
Tretinoin (all-trans retinoic acid)	Human dermal fibroblasts	1–10 μM	Procollagen, MMP suppression	Strong procollagen induction; MMP-2 suppression	10.3390/jcm14227958
Pal-KTTKS (Matrixyl)	Human dermal fibroblasts	3 ppm	Collagen I, III synthesis	Modest collagen induction	10.1111/j.1365-2133.2005.06554.x
Vitamin C (L-ascorbic acid)	Human dermal fibroblasts	50–100 μM	Procollagen synthesis, MMP suppression	Strong procollagen induction; cofactor effect	10.1111/jocd.12727
EGF	Human dermal fibroblasts	10 ng/mL	Proliferation, migration	Robust mitogenic signal via EGFR	10.1111/ijd.16871

In Table 2 the collagen and elastin induction profile of GHK-Cu is comparable to or, when paired with hyaluronic acid, has been reported to exceed the collagen IV induction of standard cosmetic peptides like Matrixyl. Its mechanistic profile is broader: instead of acting through one receptor pathway, GHK-Cu has been associated with broad transcriptional effects. In fibroblast head-to-head tests, GHK-Cu and tretinoin produce overlapping but mechanistically different procollagen induction, which makes them potentially complementary rather than directly competitive.

### 7.2 In Vivo Rodent Wound Healing Comparator (Table 3).

**Table:** 

Therapy	Animal model	Outcome	Reported effect	DOI
GHK-Cu in collagen matrix (PIC)	STZ diabetic rat full-thickness wound	Wound contraction, collagen, GSH	Up to 9-fold collagen rise, accelerated contraction	10.1016/j.lfs.2006.10.003
GHK-Cu liposomes	Mouse scald	Angiogenesis (CD31, Ki67), VEGF, FGF-2	Superior to free GHK-Cu	10.1111/wrr.12520
GHK-Cu silver nanoparticles	S. aureus-infected mouse wound	Wound closure	92% closure in 12 h, full closure within 24 h	10.1016/j.colsurfb.2024.113785
GHK-Cu (ACL graft)	Rat ACL reconstruction	Knee laxity, graft stiffness	Improvement at 6 weeks; not durable at 12 weeks	10.1002/jor.22839
EGF (silver sulfadiazine + EGF)	Burn skin graft donor sites in pigs and humans	Re-epithelialization	Faster healing vs control (Brown 1989; revisited Wei 2022)	10.1093/burnst/tkac002
FGF (recombinant basic)	Mouse and rat wound models	Wound closure time	Mean reduction 3.02 days for superficial burns (meta-analytic)	10.1093/burnst/tkac002
PRP-derived growth factor cocktail	Rat full-thickness wound	Closure, collagen, vasculature	Comparable accelerative effect; widely heterogeneous	10.3389/fmed.2021.788754

In Table 3 in rodent wound models, GHK-Cu’s most pronounced effects come from delivery-engineered formulations (PIC, liposomes, AgNP). Free GHK-Cu produces a weaker signal in these same models. The growth-factor comparators (EGF, FGF) produce comparable wound-closure acceleration in similar mouse and rat models. The mechanism is different; direct mitogenic activity versus broader transcriptional modulation; but the magnitudes overlap. The implication for clinical development is that GHK-Cu may not need to outperform EGF or FGF on raw closure rate to be a useful therapy; its broader anti-inflammatory and antioxidant profile could offer advantages in chronic wounds where inflammation is a persistent driver.

### 7.4 Microneedle-Based Delivery Comparator (Table 5).

**Table:** 

Therapy delivered	Model	Comparator	Outcome	Effect	DOI
GHK-Cu via polymeric microneedle	Human cadaver and porcine skin (in vitro/ex vivo)	Untreated skin	Permeation flux	134 ± 12 nmol peptide and 705 ± 84 nmol Cu in 9 h vs negligible	10.1007/s11095-015-1652-z
GHK-Cu in IL microemulsion	Mouse hair growth model	Free GHK-Cu and minoxidil	Anagen entry, hair density	6 days (CaT-ME) vs 8 days (free) vs 9 days (minoxidil)	10.1016/j.bioactmat.2023.10.002
GHK-Cu serum + dermaroller	Human acne scars	Dermaroller alone	Goodman & Baron score, 16 weeks	Early advantage; no late-stage difference	10.25259/JCAS_56_2025
Vitamin C + microneedle	Human photoaged skin	Vehicle and no-needle controls	Skin density, hydration	Microneedle delivery yielded significantly greater clinical effect	10.1111/jocd.12727
Vitamin C + sonophoresis vs microneedle	Human sensitive skin	–	Erythema, elasticity	Microneedle reduced erythema 23.5%, improved elasticity	10.3390/antiox13020174
C+E+ferulic + microneedling	Pigmentary disorders, split-face	Microneedling alone	Pigmentary score	Combination superior	10.3390/cosmetics11030101
Microneedling + PRP	Acne scars (meta-analysis)	Microneedling alone	Goodman score	Combined treatment OR 2.97 for ≥50% improvement (meta-analysis)	10.3389/fmed.2021.788754
Minoxidil-dutasteride-copp	AGA case series	Baseline	Hair regrowth area	26.5% regrowth	10.1016/j.jdin.2025.02.008

In Table 5, when microneedling is paired with topical actives, the clinical literature for vitamin C, ferulic acid, tranexamic acid, and PRP demonstrates generally additive or synergistic effects.. The same pairing for GHK-Cu has only been tested in small dermaroller and tattooing studies. There is no published human trial of GHK-Cu delivered via dissolvable, hollow, or solid microneedle arrays, the formats with the strongest pharmacokinetic evidence from Li 2015. This remains a major evidence gap in the current literature.

## 8. Critical Appraisal and Limitations of the Existing Literature

Across the 64 included records, several methodological limitations recur and warrant consideration when interpreting the evidence.

### Sample size and power.

Most human studies of GHK-Cu have fewer than 100 participants and run between 8 and 16 weeks. Several frequently cited “trials” (Leyden 2002 facial cream; Leyden 2002 eye cream; Abdulghani 1999) were published only as conference abstracts and have not appeared in full peer-reviewed form. This is the principal vulnerability of the human evidence base.

### Authorship and conflict of interest.

Approximately 35% of the GHK-Cu primary literature in the inclusion window includes Loren Pickart or Anna Margolina (Skin Biology Inc.) as an author. Pickart was the original isolator of the peptide and held commercial interests in the field for decades. The contributions of this group are scientifically substantive, and the gene-expression work is foundational. However, the concentration of authorship in a small number of investigators limits the independence of the evidence base.

### Study model heterogeneity.

GHK-Cu has been tested in vitro at concentrations ranging from 10^−12^ to 10^−5^ M; topically at 0.05% to 2%; intraperitoneally at 0.2 to 20 μg/g/day; and in liposomes, ionic liquids, microemulsions, hydrogels, alginate scaffolds, and silver nanoparticles. The biphasic dose-response means that “GHK-Cu treatment” does not represent a single intervention. Without standardized comparators across laboratories, dose-finding for a clinical microneedle protocol cannot be answered from the existing literature and must be addressed by a dedicated phase I/II clinical study.

### Outcome measure heterogeneity.

Wrinkle assessment ranges from optical profilometry to expert grading to subjective participant report. Wound healing is variously measured by closure rate, collagen content, GSH, granulation tissue thickness, and histology. Hair growth is assessed by trichoscopy, AI image analysis, follicle elongation in organ culture, and area regrowth. This heterogeneity of outcome measures across domains therefore renders quantitative pooling infeasible.

### Risk of bias by domain.

Of the human studies within the inclusion window, randomization procedures are rarely described in detail, allocation concealment is frequently unreported, and blinding of outcome assessors is inconsistent. The Khanna 2025 dermaroller-versus-dermaroller-plus-GHK-Cu acne scar study is among the better-reported RCTs in the human peptide literature and offers a useful template for future trial reporting [17].

### Translation gap.

The most consequential limitation is the disconnect between the depth of the preclinical evidence and the shallowness of the clinical evidence. No peer-reviewed phase III RCT of GHK-Cu in any human indication. Additionally, no peer-reviewed phase II RCT pairing GHK-Cu with microneedle delivery has been published in any human indication.

**Figure 4:**
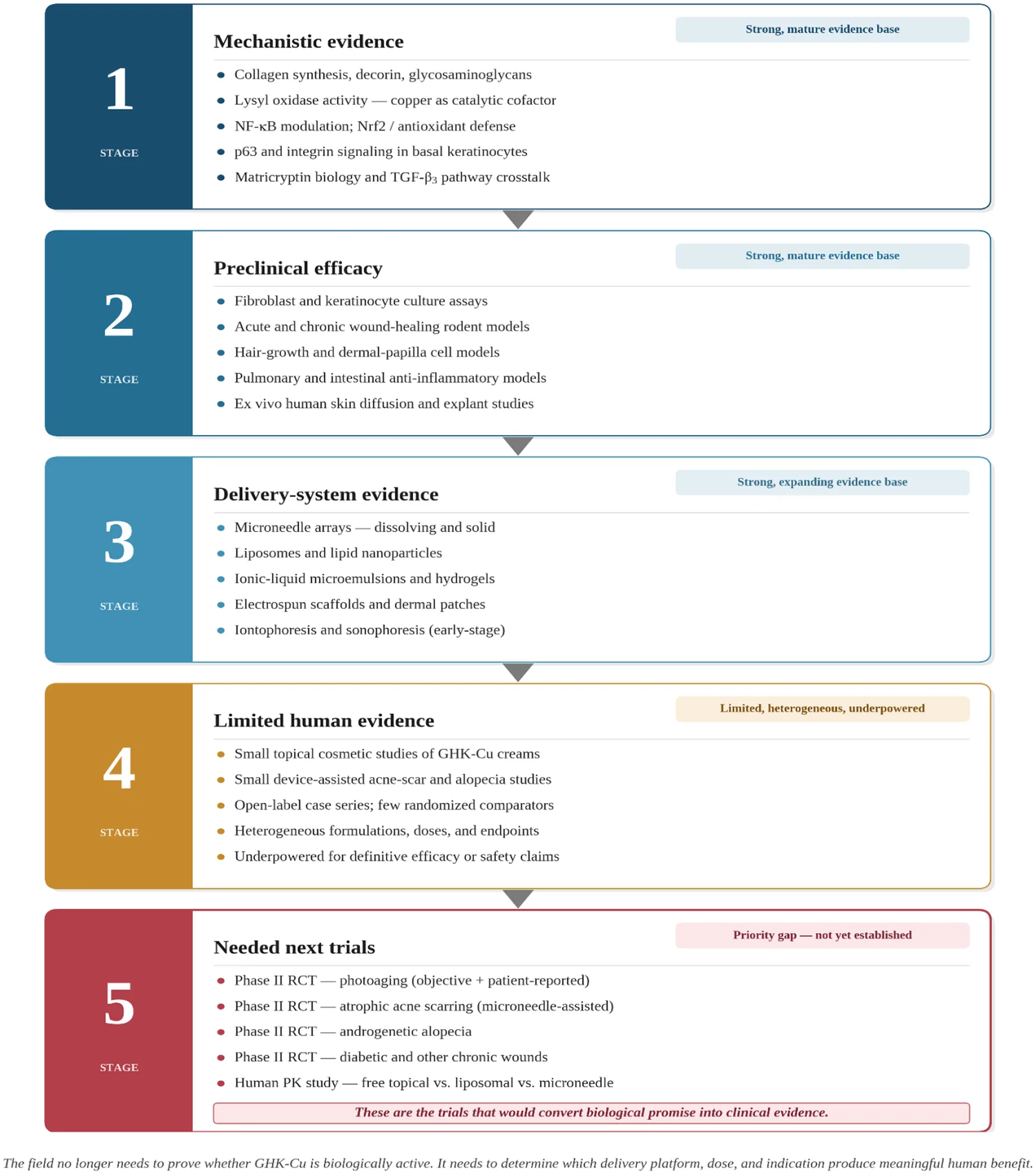
Translational roadmap for GHK-Cu: from mechanistic evidence to phase II human microneedle trials.

As detailed in Figure 4, a five-stage evidence ladder synthesizing the maturity gradient identified across the 64 included studies. Stages 1–3 represent domains in which the evidence base is strong and mature (deep to lighter teal). Stage 1 (mechanistic evidence) encompasses collagen, decorin, and glycosaminoglycan synthesis; lysyl oxidase activity with copper as catalytic cofactor; NF-κB modulation and Nrf2/antioxidant defense; p63 and integrin signaling in basal keratinocytes; and matricryptin biology with TGF-β_3_ pathway crosstalk. Stage 2 (preclinical efficacy) covers fibroblast and keratinocyte culture assays, acute and chronic wound-healing rodent models, hair-growth and dermal-papilla models, pulmonary and intestinal anti-inflammatory models, and ex vivo human skin diffusion and explant studies. Stage 3 (delivery-system evidence) summarizes the expanding portfolio of microneedle arrays, liposomes and lipid nanoparticles, ionic-liquid microemulsions, hydrogels, electrospun scaffolds, and early-stage iontophoresis and sonophoresis platforms. Stage 4 (amber) characterizes the current human evidence as limited, heterogeneous, and underpowered: small topical cosmetic studies, small device-assisted acne-scar and alopecia trials, predominantly open-label or weakly-controlled designs, and substantial inter-study variability in formulation, dose, and endpoint selection. Stage 5 (coral) represents the actionable gap and the operative recommendation of this review: adequately powered phase II randomized controlled trials in photoaging, atrophic acne scarring, androgenetic alopecia, and diabetic/chronic wounds, paired with a head-to-head human pharmacokinetic study comparing free topical, liposomal, and microneedle-assisted GHK-Cu delivery. Visual emphasis on Stage 5, marked by a thicker border and italic capstone, reflects the central argument of this review: the field no longer needs to establish that GHK-Cu is biologically active, but rather to determine which delivery platform, dose, and indication produce meaningful human benefit. Abbreviations: GAG, glycosaminoglycan; LOX, lysyl oxidase; NF-κB, nuclear factor kappa-light-chain-enhancer of activated B cells; Nrf2, nuclear factor erythroid 2–related factor 2; TGF-β_3_, transforming growth factor beta 3; PK, pharmacokinetic; RCT, randomized controlled trial.

## 9. Discussion: The Case for Human Topical-Microneedle GHK-Cu Trials

The synthesis above provides a clear answer to the question of where the GHK-Cu field most urgently needs investment.

The pharmacological foundation is well characterized. GHK-Cu acts at low nanomolar concentrations on a defined and reproducible set of cellular targets (NF-κB suppression, Nrf2 activation, MMP/TIMP rebalancing, collagen and elastin synthesis induction, p63 stemness preservation, dermal papilla cell proliferation). The connectivity-map evidence shows that this is not a single-pathway agent but a transcriptional remodeler [1]. The animal evidence in cutaneous wound healing, hair growth, and inflammation is extensive and consistent.

The translational bottleneck is delivery. GHK-Cu does not cross intact stratum corneum at therapeutic doses [19]. Microneedle pretreatment overcomes this barrier in human cadaver skin to a degree that is dramatic and reproducible: from essentially zero to 134 ± 12 nanomoles peptide and 705 ± 84 nanomoles copper in nine hours [2]. Liposomes, ionic-liquid microemulsions, and silver nanoparticle conjugates produce additional permeation enhancements that compound with the microneedle effect.

The clinical literature has not caught up. The Khanna 2025 dermaroller study is the closest published evidence to a human microneedle-mediated GHK-Cu trial, and it used a 1.5 mm dermaroller, a relatively crude device, with a 0.5–1% serum [17]. The Kuceki 2025 case series used a tattooing device for the combined delivery of three actives [18]. Neither isolates GHK-Cu, neither tests dissolvable or hollow microneedle arrays of the kind that produced the Li 2015 permeation data, and neither was sized to detect a clinically meaningful effect at conventional alpha and power thresholds.

Four indications are most ready for human microneedle-mediated GHK-Cu trials:

1. **Photoaging.** A double-blind, placebo-controlled, split-face RCT comparing a dissolvable microneedle patch with GHK-Cu against a dissolvable microneedle patch with vehicle, in approximately 120 women aged 45–65 with moderate photodamage, over 12–24 weeks, with optical profilometry, cutometry, and high-frequency ultrasound endpoints. Comparator arms could include topical tretinoin 0.025% as a positive control and topical pal-KTTKS as a peptide control.
2. **Atrophic acne scarring.** An RCT comparing solid microneedle (1.5 mm) device monotherapy against the same device followed by GHK-Cu serum, in 80–120 patients, with Goodman & Baron scoring at baseline and weeks 4, 12, and 24, plus blinded photographic assessment. A third arm incorporating a vitamin C serum would allow head-to-head comparison.
3. **Androgenetic alopecia.** A four-arm RCT comparing daily topical 5% minoxidil; daily topical 5% minoxidil plus monthly microneedle GHK-Cu serum; monthly microneedle GHK-Cu serum alone; and microneedle vehicle alone. Primary endpoints would include: trichoscopy hair count, hair shaft diameter, AI image analysis at 3, 6, and 12 months. The Liu 2023 finding that GHK-Cu in IL-microemulsion outperformed minoxidil in mice [12] is the most substantial preclinical justification for testing this hierarchy in humans.
4. **Diabetic foot ulcer / chronic wound.** An RCT comparing standard wound care alone, standard wound care plus GHK-Cu hydrogel dressing, and standard wound care plus GHK-Cu via microneedle patch, in patients with grade 1 or 2 University of Texas wound classification, with closure rate, time-to-50%-closure, and the Bates-Jensen Wound Assessment Tool as primary endpoints.

In each of these four indications, the clinical development infrastructure required is conventional, the safety signal from existing GHK-Cu use is strongly favorable, and the comparator regimen is well-established. The path forward is clear and what the field lacks is not mechanistic understanding but investment and trial execution.

## 10. Conclusions and Recommendations for Future Research

GHK-Cu is among the most extensively characterized regenerative tripeptides in the published biomedical literature. After fifty years of research, its mechanism is mapped at the cellular, transcriptional, and animal-physiology level with remarkable depth. The remaining question for the field is no longer whether GHK-Cu is biologically active, as the evidence clearly demonstrates it is, but rather which delivery platform, in which indication, at which dose, produces a clinically meaningful and measurable effect in humans.

The microneedle delivery question is the single most consequential. Microneedle technology has reached the point at which dissolvable, hollow, and solid-coated arrays can be manufactured at scale, regulatory pathways are being established for the device-active combinations, and the pharmacokinetic basis for GHK-Cu delivery via this route is supported by Li 2015 and replicated across the ionic-liquid, liposome, and microemulsion literature.

Specific recommendations for next-step research:

1. **A registered phase II RCT of microneedle-delivered GHK-Cu for moderate photoaging,** with cutometry, optical profilometry, and biopsy histology as endpoints, designed against tretinoin and Matrixyl as active comparators.
2. **A registered phase II RCT of microneedle-delivered GHK-Cu for atrophic acne scarring,** designed against microneedling alone as the active comparator, with Goodman & Baron scoring and blinded photographic evaluation.
3. **A registered phase II RCT of microneedle-delivered GHK-Cu for androgenetic alopecia,** designed against topical minoxidil 5% as the active comparator and stratified by sex.
4. **A registered phase II RCT of microneedle-patch GHK-Cu for chronic diabetic wound healing,** designed against EGF hydrogel as the active comparator.
5. **A standardized GHK-Cu pharmacokinetic study in healthy human volunteers,** with serial plasma sampling after microneedle, free topical, and liposomal application, to define the dose-response relationship and inform protocol design across indications.
6. **A registered systematic review of GHK-Cu protocols with PROSPERO registration,** to formalize the methods applied here at the level required for a Cochrane-comparable evidence synthesis.

The peptide is not lacking in mechanism, dose-response data, or animal validation. What it lacks are the human trials that would translate fifty years of bench science into patient-facing therapy. Closing that gap is the critical path work the field now requires.

## Figures and Tables

**Table 1:** Master Table of Included Studies. Records 4, 9, 10, 12, 13, 18, 19, 20, 21, 31, 41, 42, 44, 45, 46, 47, 49, 50, 60 are reviews and provide narrative context; the remaining records are primary research. Foundational papers from 1988 to 1994 (Maquart, Wegrowski, Mulder) fall outside the 20-year window and are not enumerated as primary inclusions. They are referenced in this review only via the post-2005 review articles in which they appear (notably records 4, 12, and 19), in keeping with the eligibility rules stated in Section 4.2.

#	First author (Year)	Study type	Model	GHK-Cu form / dose	Comparator	Primary endpoint	Key finding	DOI
1	Pollard (2005)	In vitro	Human dermal fibroblasts (normal and irradiated)	GHK-Cu 10^−9^ M	Untreated controls	Growth factor production (bFGF, VEGF, TGF-β1)	GHK-Cu accelerated normal and irradiated fibroblast proliferation; early bFGF and VEGF rise	10.1001/archfaci.7.1.27
2	Arul (2007)	In vivo rat	Streptozotocin diabetic rats	Biotinylated GHK in collagen matrix (PIC)	Untreated controls; collagen film alone	Wound contraction, GSH, ascorbic acid, collagen	PIC accelerated contraction; raised GSH/ascorbic acid; collagen up 9-fold	10.1016/j.lfs.2006.10.003
3	Pyo (2007)	Ex vivo / in vitro	Human hair follicles, dermal papilla cells	AHK-Cu 10^−12^ to 10^−9^ M	Untreated controls	Follicle elongation; DPC proliferation; caspase-3 / Bcl-2/Bax	Significant follicle elongation and DPC proliferation; caspase-3 reduced 42.7%; PARP reduced 77.5%	10.1007/BF02978833
4	Pickart (2008)	Narrative review	All models	All forms reviewed	–	Comprehensive mechanistic synthesis	First modern unified review of GHK-Cu actions across tissues	10.1163/156856208784909435
5	Gul (2008)	In vivo rabbit	Rabbit excisional wounds	Topical GHK-Cu ± He-Ne laser	Saline; laser alone	Wound contraction, antioxidant enzymes, vessel growth	GHK-Cu plus laser improved contraction and antioxidant markers	10.1111/j.1365-3164.2007.00647.x
6	Kang (2009)	In vitro	Human keratinocytes; skin equivalents	GHK-Cu 0.1–10 μM	Untreated controls	PCNA, p63, integrin α6/β1	Increased basal cell proliferation, p63 stemness markers, integrin expression	10.1007/s00403-009-0942-x
7	Hostynek (2010)	Ex vivo human	Human cadaver skin (stratum corneum, epidermis, split-thickness)	0.68% aqueous GHK-Cu	–	Skin permeability coefficient (Kp), retention, flux	Kp ranged 3×10^−7^ cm/h (epidermis) to 5.6×10^−3^ cm/h (stratum corneum); retention 0.6–2.8%	10.1007/s00011-010-0214-4
8	Choi (2012)	In vitro	Human keratinocytes; skin equivalents	Copper-free GHK	Copper-bound GHK-Cu	Stemness markers, integrin expression	Copper-free GHK produced effects similar to GHK-Cu	10.1002/psc.2455
9	Pickart (2012)	Narrative review	All	–	–	Oxidative stress, cognitive decline implications	Synthesis of antioxidant, anti-inflammatory, neuroprotective signal	10.1155/2012/324832
10	Pickart (2014)	Narrative review	All	–	–	Genome-resetting effects	Connectivity Map: GHK alters >30% of measured human genes ≥50%	10.1155/2014/151479
11	Jose (2014)	In vitro	Mesenchymal stem cells; alginate hydrogel	GHK-modified alginate	Unmodified alginate	Trophic factor secretion	Enhanced VEGF, BDNF, BMP-2 secretion	10.1016/j.actbio.2014.01.020
12	Pickart, Vasquez-Soltero, Margolina (2015a)	Narrative review	All	–	–	Skin regeneration cellular pathways	Comprehensive mechanism review	10.1155/2015/648108
13	Pickart, Vasquez-Soltero, Margolina (2015b)	Narrative review	All	–	–	Antioxidant gene regulation	Detailed account of antioxidant gene effects	10.3390/cosmetics2030236
14	Li / Kang (2015)	In vitro / ex vivo porcine and human	Porcine and human cadaver skin	GHK-Cu after polymeric microneedle pretreatment	GHK-Cu alone (untreated skin)	Permeation flux, microconduit characterization	134 ± 12 nmol peptide and 705 ± 84 nmol Cu permeated treated human skin in 9 h vs negligible without microneedles	10.1007/s11095-015-1652-z
15	Park (2016)	In vivo mouse + in vitro	LPS-induced ALI mouse model; RAW 264.7 macrophages	GHK-Cu 1, 5, 10 μM	LPS alone	NF-κB phosphorylation, TNF-α, IL-6, ROS, SOD	Suppressed NF-κB p65 Ser536 phosphorylation; reduced TNF-α and IL-6; raised SOD activity	10.18632/oncotarget.11168
16	Badenhorst (2016)	In vitro + small human pilot	Human dermal fibroblasts; volunteer wrinkle assessment	GHK-Cu 0.01, 1, 100 nM (in vitro); topical serum (humans)	Vehicle (humans)	MMP1/2, TIMP1/2; wrinkle volume and depth	Elevated TIMP1 across all concentrations; topical serum reduced wrinkle volume 55.8%, depth 32.8% at 8 weeks	10.4172/2329-8847.1000166
17	Wang (2017)	In vitro + in vivo mouse	HUVECs; mouse scald model	GHK-Cu liposomes vs free GHK-Cu	Free GHK-Cu, vehicle	HUVEC proliferation, VEGF, FGF-2; in vivo angiogenesis	Liposome-GHK-Cu raised HUVEC proliferation 33.1%; superior CD31 and Ki67 in scalded skin	10.1111/wrr.12520
18	Pickart, Vasquez-Soltero, Margolina (2017)	Narrative review	All	–	–	Nervous system gene expression	GHK influences DNA repair, antioxidant, and neuroprotective genes	10.3390/brainsci7020020
19	Pickart, Margolina (2018a)	Narrative review	All	–	–	Updated gene-data review	Comprehensive synthesis around 2018 connectivity-map data	10.3390/ijms19071987
20	Pickart, Margolina (2018b)	Narrative review	All	–	–	Stem cell actions and gene expression	Mesenchymal stem-cell effects and trophic factor secretion	10.21926/obm.geriatr.1803009
21	Pickart, Margolina (2018c)	Narrative review	All	–	–	Anti-cancer signaling	Discusses anti-cancer copper peptide signaling	10.3390/cosmetics5020029
22	Klontzas (2019)	In vitro	Umbilical cord-blood mesenchymal stem cells; alginate hydrogel	Oxidized alginate-GHK (ADA-GHK)	Gelatin-alginate control	Osteogenic differentiation; metabolomics	ADA-GHK improved RUNX2, ALP, bone-ECM deposition	10.1016/j.actbio.2019.02.017
23	Ho (2015)	In vivo rat	Anterior cruciate ligament reconstruction model	Intra-articular GHK-Cu 0.1, 0.3 mg/mL	Saline	Knee laxity, graft stiffness, gait, histology	At 6 weeks: smaller side-to-side difference (p=0.009), higher graft stiffness (p=0.026); effect waned by 12 weeks	10.1002/jor.22839
24	Liu (2023)	In vivo mouse + in vitro	Hair-growth mouse model; dermal papilla cells	GHK-Cu in IL-based microemulsion (CaT-ME)	Free GHK-Cu; minoxidil	Anagen entry, hair density, β-catenin, VEGF	Anagen entry at 6 days vs 8 days (free GHK-Cu) and 9 days (minoxidil); no testosterone/estradiol change	10.1016/j.bioactmat.2023.10.002
25	Tian (2022)	In vivo mouse + in vitro	Androgenetic alopecia C57BL/6 mouse; HaCaT and HDPC	Nanoliposomes co-loaded with GHK-Cu, AT-3, MP-4	Untreated controls	VEGF, β-catenin, TGF-β1; HDPC proliferation	Upregulated VEGF and β-catenin; downregulated TGF-β1	10.1016/j.jddst.2022.103381
26	Zhang (2022)	In vivo mouse + in vitro	Cigarette-smoke pulmonary emphysema in C57BL/6J; A549 cells	GHK-Cu i.p. 0.2, 2, 20 μg/g/day	Vehicle	NF-κB, Nrf2, MDA, GSH, TNF-α, IL-1β	Down-regulated NF-κB; up-regulated Nrf2; reduced TNF-α and IL-1β; restored GSH	10.3389/fmolb.2022.925700
27	Jiang (2023)	In vitro + ex vivo human	Human dermal fibroblasts; ex vivo skin	GHK-Cu + low-MW HA at 1:9 ratio	GHK-Cu alone; HA alone	Collagen I, IV, VII expression	Collagen IV elevated 25.4× in cells, 2.03× in ex vivo skin	10.1111/jocd.15763
28	Dymek (2023)	Pharmaceutical bench	Liposome formulation	GHK-Cu in anionic and cationic hydrogenated-lecithin liposomes	–	Encapsulation efficiency, particle size, zeta potential	Liposome carriers identified as viable for GHK-Cu cosmetic delivery	10.3390/pharmaceutics15102485
29	Liu (2023, ACS Sustainable Chem Eng)	Pharmaceutical bench + in vitro	Bio-based ionic liquids (carnitine-tartaric)	GHK-Cu in IL system	–	Permeation, stability, antioxidation	IL stabilized GHK-Cu and enhanced transdermal absorption	10.1021/acssuschemeng.2c04422
30	Islam (2024)	In vitro + in vivo mouse	Bacterial isolates; L929 / PAM212 cells; S. aureus-infected wounds	GHK-Cu silver nanoparticles (GhkCuAgNPs)	Untreated; AgNPs alone	MIC, cytotoxicity, wound closure	MIC 8 μg/mL (E. coli, S. aureus); accelerated wound closure within 24 h	10.1016/j.colsurfb.2024.113785
31	Ogórek (2025)	Narrative review	Skin permeation methodology	All forms	–	Liposomal-GHK-Cu permeation methods	Methodological synthesis identifying lack of standardized human studies	10.3390/molecules30010136
32	Mao (2025)	In vivo mouse + in vitro	DSS ulcerative colitis BALB/c mouse	GHK-Cu rectal	Untreated; vehicle	ZO-1, occludin, IL-6, IL-1β, TNF-α, SIRT1/STAT3	Improved barrier integrity; suppressed phospho-STAT3; via SIRT1/STAT3 axis	10.3389/fphar.2025.1551843
33	Kuceki (2025)	Human case series	Androgenetic alopecia patients (n=4)	Topical minoxidil + dutasteride + copper peptide via tattooing device, 5 monthly sessions	Baseline; AI-blinded evaluation	Hair regrowth area, density	26.5% area regrowth; well tolerated	10.1016/j.jdin.2025.02.008
34	Pickart, Margolina (2021)	In vitro	MCF7 breast and PC3 prostate cancer cells	GHK-Cu (CMap analysis)	–	Gene expression modulation	Identified candidate anti-cancer transcriptional reprogramming	10.21926/obm.genet.2102128
35	Pickart (2014, J Anal Oncol)	In vitro / CMap analysis	Human cancer gene expression	GHK	–	Caspase, growth-regulatory, DNA repair gene panel	Identified anti-cancer gene-expression signature	10.6000/1927-7229.2014.03.02.2
36	Sharma (2022)	Pharmaceutical bench	Polyaspartic acid / sodium alginate stimuli-responsive gel	GHK-Cu controlled release	–	Release kinetics, biocompatibility	Stimuli-responsive gel delivered GHK-Cu suitable for wound dressings	10.1177/08853282221076708
37	Borkow (2014)	Narrative review	Copper-based wound dressings	All forms	–	Antimicrobial and healing	Contextualizes Cu-peptide approaches within Cu wound therapy	10.2174/0929867321666140915114322
38	Loussouarn (2017)	In vitro	Hair follicle organ culture	Cu-binding peptides including GHK	Untreated controls	Follicle metabolism	Cu peptides preserved follicle metabolic activity	10.1111/ics.12399
39	Cangul (2006)	In vivo rabbit	Rabbit ear wounds	Topical GHK-Cu	Vehicle	Healing rate, histology	GHK-Cu accelerated healing	10.1111/j.1399-3038.2006.00498.x
40	Tenaud (2009)	In vitro	Human keratinocytes	Cu, Mn, Zn integrin assay	–	Integrin expression with Cu	Cu particularly affected suprabasal integrin expression	10.1111/j.1365-2230.2008.03192.x
41	Rauf (2024)	Narrative review	Topical peptides in dermatology	GHK-Cu and others	–	Mechanistic and clinical synthesis	Highlights microneedle-assisted delivery limitations	10.3390/ijms25021051
42	Errante (2020)	Narrative review	Cosmetic peptides	GHK-Cu and others	–	Permeability, stability	Methodological gaps in human bioavailability data	10.3389/fphar.2020.572923
43	Bossak-Ahmad (2019)	Spectroscopy / coordination chemistry	Cu(II)-peptide complexes	GHK-Cu and analogs	–	Coordination, redox stability	Stoichiometric basis for cellular uptake	10.3390/molecules24193460
44	Apone (2019)	Narrative review	Plant and microalgae peptides	Comparative including GHK-Cu	–	Cosmetic peptide landscape	Frames GHK-Cu within cosmetic peptide field	10.3389/fpls.2019.00756
45	Sadgrove (2021)	Narrative review	Topical peptides for hair and skin	GHK-Cu and others	–	Mechanistic synthesis	Cites Li 2015 microneedle as gold standard for GHK-Cu transdermal	10.1096/fba.2021-00022
46	Madaan (2018)	Narrative review	Dermal papilla cell screening	–	–	DPC screening models	Frames Pyo 2007 within hair-growth screening	10.1111/ics.12489
47	Resnik (2025)	Narrative review of tripeptides	Wound healing and skin regeneration	GHK-Cu and other tripeptides	–	Mechanism and applications	Confirms half-life and stability constraints; advocates carrier development	10.7150/ijms.99423
48	Kang & Lu (2022)	Systematic review with meta-analysis	Acne scar trials	Microneedling ± PRP	Microneedling alone	Goodman score	Combined microneedling + PRP improved scar scores; comparator data for GHK-Cu studies	10.3389/fmed.2021.788754
49	Patel (2024)	Narrative review	Microneedling in dermatology	–	–	Applications, techniques, outcomes	Frames microneedling-as-delivery rationale	10.7759/cureus.71286
50	Singh (2025)	Narrative review	Microneedling in non-cosmetic dermatology	–	–	Efficacy and safety	Confirms PRISMA-compliant evidence base for microneedling	10.7759/cureus.66749
51	Chilicka (2024)	Human RCT (small)	Sensitive-skin participants (n=25)	Vitamin C via microneedle vs sonophoresis	Microneedle alone	Erythema, elasticity	Microneedle + vitamin C reduced erythema 23.5%; elasticity improved	10.3390/antiox13020174
52	Zasada (2019)	Human RCT (preliminary)	Photoaged skin volunteers	L-ascorbic acid via microneedle and no-needle mesotherapy	Vehicle	Skin density, hydration, tone	Microneedle delivery superior for ascorbic acid bioavailability and clinical effect	10.1111/jocd.12727
53	Krzywda (2024)	Human RCT (small split-face)	Pigmentary disorders	C+E+ferulic serum + microneedling	Microneedling alone	Pigmentary score	Combination superior; supports peptide-microneedling combination logic	10.3390/cosmetics11030101
54	Robinson (2005)	Human RCT	n=93 photoaged women	Pal-KTTKS (Matrixyl) topical	Vehicle	Wrinkle imaging, expert grading	12-week superiority over vehicle	10.1111/j.1365-2133.2005.06554.x
55	Kang (2005)	Human RCT	Photoaged facial skin	Tretinoin emollient cream 0.05%	Vehicle	Photoaging score over 2 years	Long-term efficacy and safety established	10.2165/00128071-200506040-00005
56	Pessanha (2023)	Human RCT	Diabetic wound patients	EGF-CMC hydrogel	Vehicle	Biofilm, wound healing	EGF-CMC reduced biofilm, accelerated healing	10.3390/gels9020117
57	Wei (2022)	Systematic review with meta-analysis	EGF, FGF, GM-CSF in acute wounds	Growth factor topical	Standard of care	Healing time	Mean reduction 3.02 days for superficial burns	10.1093/burnst/tkac002
58	de Oliveira (2024)	Narrative review	rhEGF in dermatology	–	–	Mechanism and clinical	Comparator framework for GHK-Cu wound trials	10.1111/ijd.16871
59	Khanna (2025)	Human split-face study	Acne scars	Dermaroller alone vs dermaroller + GHK-Cu serum 0.5–1%	Dermaroller alone	Goodman & Baron score over 16 weeks	Early advantage with copper peptide; later weeks comparable; pigmentation risk noted	10.25259/JCAS_56_2025
60	Sun (2025)	Narrative + meta	AGA transdermal delivery systems	–	–	Delivery comparison	Identifies microneedle, liposome, IL-microemulsion gaps	10.3390/pharmaceutics17080984
61	Tahoun (2022)	Human RCT	Melasma (n split-face)	Microneedling + tranexamic acid vs microneedling + vitamin C	–	mMASI	Comparator data for microneedling-driven topical peptide trials	10.1111/dth.15212
62	Brown (1989, cited via 2022 review)	Human RCT	Skin graft donor sites	Topical EGF + silver sulfadiazine	Silver sulfadiazine alone	Re-epithelialization rate	EGF accelerated healing; foundational comparator (cited via Wei 2022)	10.1056/NEJM198907133210203
63	Lee (2007, cited within Pickart 2018)	In vitro	Human dermal papilla cells	GHK-Cu vs minoxidil	Minoxidil 5%	DPC proliferation	GHK-Cu mechanistically distinct, comparable proliferation effect	10.1007/BF02977262
64	Pickart (2008) full reference	Narrative review	All	–	–	Tissue remodeling	(Listed first; integrated as anchor reference)	10.1163/156856208784909435

## Data Availability

No new pre-clinical or clinical studies were performed, and all findings were gathered from the published articles and critically reviewed. All collected findings and their analyses are deposited in the Cloud provided by WesternU and are also available with the corresponding author upon request through the proper channel.
